# Using Directly Observed Therapy (DOT) for latent tuberculosis treatment – A hit or a miss? A propensity score analysis of treatment completion among 274 homeless adults in Fulton County, GA

**DOI:** 10.1371/journal.pone.0218373

**Published:** 2019-06-21

**Authors:** Udodirim Onwubiko, Kristin Wall, Rose-Marie Sales, David P. Holland

**Affiliations:** 1 Medical and Preventive Services, Fulton County Board of Health, Atlanta, Georgia, United States of America; 2 Department of Epidemiology, Rollins School of Public Health, Emory University, Atlanta, Georgia, United States of America; 3 Tuberculosis Program, Georgia Department of Public Health, Atlanta, Georgia, United States of America; 4 Division of Infectious Diseases, School of Medicine, Emory University, Atlanta, Georgia, United States of America; UMDNJ-New Jersey Medical School, UNITED STATES

## Abstract

Latent tuberculosis infection (LTBI) treatment in persons at increased risk of disease progression is a key strategy with the strong potential to increase rate of tuberculosis (TB) decline in the United States. However, LTBI treatment in homeless persons, a population at high-risk of active TB disease, is usually associated with poor adherence. We describe the impact of using directly observed treatment (DOT) versus self-administered treatments (SAT) as an adherence-improving intervention to administer four months of daily rifampin regimen for LTBI treatment among homeless adults in Atlanta. Retrospective analysis of clinical care data on 274 homeless persons who initiated daily rifampin treatment for LTBI treatment at a county health department between January 2014 and December 2016 was performed. To reduce bias from non-random assignment of treatment, an inverse probability of treatment weighted (IPTW) logistic regression model was used to assess the effect of treatment type on treatment completion. Subgroup analyses were performed to assess heterogeneity of treatment effect on LTBI completion. Of 274 LTBI treatment initiators, 177 (65%) completed treatment [DOT 118/181 (65%), SAT 59/93 (63%)]. In the fully adjusted and weighted analysis, the odds of completing LTBI treatment on DOT was 40% higher than the odds of completing treatment by SAT [adjusted odds ratio (95% CI), aOR: 1.40 (1.07, 1.82), p = 0.014]. The unstable nature of homeless persons’ lifestyle makes LTBI treatment difficult for many reasons. Our study lends support to the use of DOT to improve LTBI treatment completion among subgroups of homeless persons on treatment with daily rifampin.

## Introduction

Tuberculosis (TB) is one of the top ten leading causes of death globally.[1] In 2015, about 1.4 million deaths were attributed to TB, making it the global leading cause of death from an infectious disease, ahead of HIV/AIDS.[2] Using strategic disease surveillance and prioritized TB control methods, the current incidence of TB in the United States (US) has been driven to the lowest in recorded history. [3] The role that implementation of the top three priorities of TB control (early identification of active TB cases, prompt initiation of treatment with recommended regimen and the identification with treatment of exposed contacts) has played in driving the decline in active TB in the US has been well documented.[3,4] Epidemiologic modeling, however, has shown that achieving the goal of TB elimination well before the end of this century in all at-risk populations in the US will require a stronger focus on identification and treatment of latent TB infection (LTBI) among persons at high-risk of TB disease.[3–5] These high-risk groups include HIV-infected persons, immigrants and refugees from countries with high TB burden, alcohol and drug abusers and persons dwelling in congregate settings such as incarceration centers and homeless housing facilities.[6,7] With four-fifths (80%) of all active TB disease in the US attributed to reactivation of latent TB infections (LTBI) rather than primary TB infections[8], the effective and prompt treatment of LTBI in these high risk groups remains the key strategy for eliminating TB in the US.

Within the last decade, the field of LTBI therapeutics has seen several innovative advances and research. Newer treatment regimens with shorter durations have been introduced and extensively studied for both efficacy and non-inferiority in comparison to the well-known nine months of daily isoniazid. These new regimens include four months of rifampin and three months of Isoniazid and Rifapentine.[9–13] Despite these advances, LTBI treatment adherence and completion in non-clinical trials setting, particularly among homeless persons, has remained poor.[11,14,15] A recent systematic review of 83 studies on LTBI treatment by Sandgren *et al* found that proportions of persons completing LTBI treatment among homeless persons was consistently lower (23–71%) than proportions completing treatment in other population subgroups; general population (39–96%), case contacts (48–82%), HIV positive clients (55–95%), immigrants (7–86%) and inmates (4–100%).[16]

While poor adherence to prescribed medical intervention in any population is a major hurdle in achieving desired healthcare outcomes, it is especially problematic when the health condition is a chronic illness that lacks obvious symptoms, presenting no discernable disruption to one’s quality of life.[17] Poor adherence to prescribed LTBI treatment is an important obstacle among homeless persons. This is because adherence to LTBI treatment is the key link between a latent infection and achieving the desired reduction in the risk of progression to active TB. Several adherence-improving interventions have been proposed and explored in the past. One of those explored in relation to TB disease management is directly observed therapy (DOT).[12,15,18–20] Its use in active TB management is well documented. However, DOT’s impact on LTBI treatment among homeless adults on daily rifampin therapy is unclear. We aim to fill that gap by assessing and quantifying the impact of using DOT for administration of four months daily rifampin treatment for LTBI among homeless persons.

## Methods

### Study design, data source and ethics statement

This was a retrospective observational cohort study. We abstracted demographic and routine clinical data of homeless persons who received TB screening, evaluation and LTBI treatment using 4 months of rifampin with the county health department between January 2014 and December 2016. Ethical approval was obtained from the Institutional Review Board (IRB) of Georgia Department of Public Health (Project #: 170405). All data used were fully anonymized prior to analysis and IRB waived the requirement for informed consent.

### Background on study sample

An outbreak of isoniazid-resistant TB among Fulton County’s homeless population began in 2008.[21] As at December 2017, a total of 110 TB cases in the county have been linked to this outbreak.[22] In the report by Powell *et al*, the epidemiological curve of this outbreak was described in three phases–initial phase when only one facility was involved (2008–2009), quiescence phase when cases waned (2010–2013) and the recrudescence phase when multiple facilities became involved (2014 and thereafter).[21,22] A full description of the epidemiology of the associated cases and the outcomes of the direct contact tracing are well documented in prior studies.[21,22]

Following the resurgence of the outbreak in 2014, several county-wide outbreak control measures were instituted.[21,22] One of these measures was the implementation of mandatory TB screening in all Atlanta homeless housing facilities in May 2015.[21,22] Prior to this, outbreak-related TB screening services were only provided onsite at the Fulton County health department or at sporadic intervals on mobile units sent out to the affected shelters. Acceptance of TB screening was however left to the discretion of the shelter user. With the new TB screening policy, TB screenings were made available several days every week at many homeless shelter locations, whether an outbreak-related case had been identified at the shelter or not. The screenings were also made mandatory for all persons seeking use of these facilities, irrespective of their direct contact status with a previously diagnosed outbreak TB case. Each shelter user was expected to get screened for TB within 7 days of initial shelter entry and every 6 months thereafter to guarantee continued access to any of the shelters.[22] Both onsite (TB clinic) and offsite (shelter locations) TB screenings were performed using either tuberculin skin test (TST) or an interferon gamma release assay method (QuantiFERON Gold-in-tube tests (QFT)).[21,22] Homeless persons who tested positive for TB infection either through TST (>10mm induration) or QFT were referred to the health department for clinical evaluation. Homeless persons with positive QFT or TST who presented at the TB clinic for evaluation were clinically assessed to exclude active TB disease and those diagnosed with untreated LTBI were offered preventive treatment with either four months of rifampin (4R) (if patient had over 2 weeks history of consistent homeless shelter stay) or three months of Rifapentine-Isoniazid (3HP) ((if they had lower than 2 weeks of consistent homeless shelter stay). [22]

Another outbreak control measure implemented after the resurgence was in the area of LTBI treatment among homeless persons.[22] Prior to this, all LTBI treatment were self-administered (SAT). With the support of the Georgia State Department of Public Health, the Atlanta TB taskforce instituted the use of directly observed treatment (DOT) for LTBI treatment among homeless persons undergoing treatment with the health department. DOT was provided 5 days a week for homeless persons on rifampin and once a week for those on Rifapentine-isoniazid treated persons.

This analysis was limited to homeless persons who were treated for LTBI at the county health department using rifampin between January 2014 and December 2016.

### LTBI treatment groups

Homeless persons treated by SAT were provided a pill box containing 30 doses of rifampin each month to self-administer daily. Self-reports of treatment adherence were recorded for SAT subjects at each monthly follow-up visit. Those treated by DOT met with trained disease intervention specialists each business day of the week at pre-determined locations (current shelter of residence, the park or the TB clinic) to receive their daily dose of rifampin. Assigned doses were ingested under direct observation and daily logs of successfully administered doses were maintained by the disease intervention specialists. Data on patient characteristics at treatment initiation including demographics (gender, age and race), clinical history (HIV status, self-reported social characteristics (any current alcohol use, non-alcoholic substance use (injectable and non-injectable) and mental/behavioral health-diagnosis in the past) were measured.

### Treatment outcome

The outcome of interest was treatment completion. For patients in the SAT group, treatment completion was defined as picking up all four months’ supply of rifampin within five months of treatment initiation with self-reports of complete ingestion of all doses provided at the clearance visit. For the DOT-treated group, treatment completion was defined as taking all assigned doses of rifampin (5 doses per week for 16 weeks + 6 extra doses = 86 doses) within five months of treatment initiation.

### Data analysis

Distributions of patient characteristics were calculated using frequencies and percentages. All patients treated with rifampin prior to May 2015 were treated by SAT while DOT was almost exclusively used after May 2015. Due to the very large proportion of study subjects who identified as Black or African American, all persons who were not African American (i.e. non-Hispanic whites, Hispanic/Latinos, Asians, Arabs, Native Americans, Pacific Islanders and others) were placed in a larger “Not Black” group to avoid having data counts less than 5 in some categories of race. To reduce bias due to non-random assignment to treatment group, propensity scores were used to balance the distribution of client characteristics in the treatment groups at baseline. Propensity score analysis is a statistical technique used to reduce bias resulting from non-random nature of treatment assignment seen in observational studies.[23–27] The estimated propensity scores (PS) represent the probability of treatment assignment conditional on the measured baseline covariates.[24–26] We estimated the propensity of each patient receiving the treatment type used to treat LTBI by modeling the treatment type received on all the measured baseline covariates in a logistic regression model.[25–27]. We examined the degree of overlap in the distributions of estimated propensity scores in the treatment groups (Common support) using histograms.[24,26,28]

Four methods of applying the scores in assessment of treatment effect (stratification, matching, inverse probability of treatment weighting using the propensity score, and covariate adjustment) have been described.[24–27,29,30] Among the four, weighting using inverse probability of treatment and propensity score matching have been shown to eliminate a greater degree of systematic differences between treatment arms.[29] While propensity score matching was shown to have marginally superior performance compared to weighting with inverse propensity of treatment, it requires the formation of matched sets of subjects with similar scores drawn from both treatment arms and may entail exclusion of patients when a comparable match is not available.[25,27,31,32] Given the small number of subjects in this study cohort (n = 274), we selected to use weighting with inverse probability of treatment to benefit from its superior performance in reducing confounding while retaining all observations in the analysis. The inverse of the estimated propensity scores was calculated and stabilized (to reduce variability in each group and reduce the influence of outlier weights) by multiplying the inverse of probability of treatment weights (IPTW) with the mean weights of the corresponding treatment group.[24–26,28,30] We assessed the degree to which confounding by the measure covariates have been balanced by comparing the standardized differences in the unweighted and weighted samples. Standardized difference <0.15 was used as the bench-mark to indicate balance of observed baseline characteristics after application of the weighted propensity scores.[24,27]

Independent associations between treatment completion and observed patient characteristics were assessed using unadjusted logistic regression models. The effect of modality used (DOT vs. SAT) on treatment completion was estimated using a weighted parsimonious logistic regression model (containing the main exposure alone) and also with a doubly robust logistic regression model (weighted logistic regression model that was fully adjusted for all measured covariates)[24,27,32–34]. For all statistical tests, p values <0.05 were considered to indicate significant associations. All statistical analyses were performed in SAS v 9.4 (SAS Institute, Cary, NC).

## Results

### TB screening and evaluation

**Fig 1:**
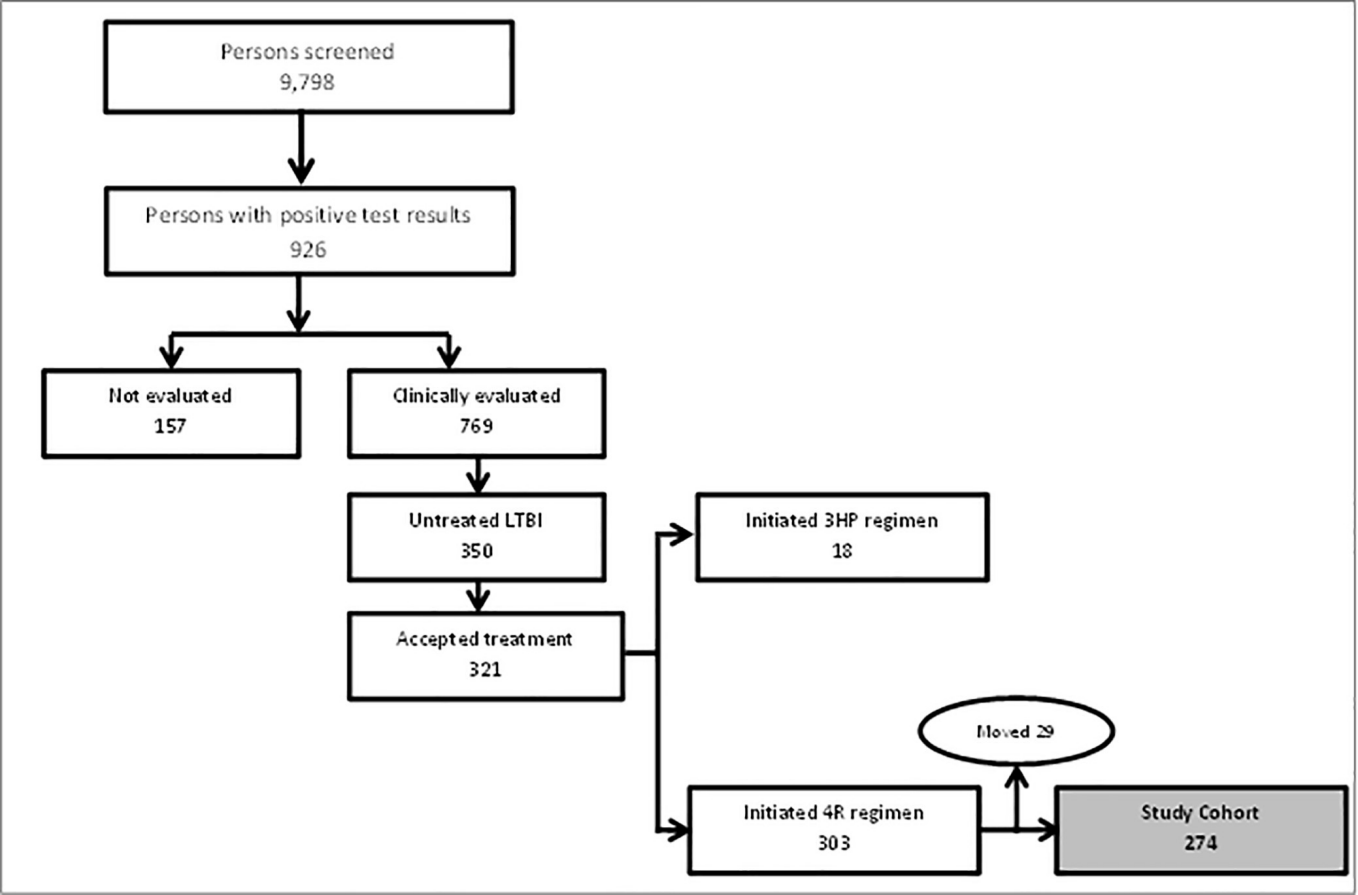
Flow chart showing study sample selection among homeless adults treated for latent TB infection, Fulton County GA 2014–2016.

A total of 13, 082 TB screening tests [TST– 9,462 (78%), QFT– 3,620 (28%)] were performed among 9,798 homeless persons between January 2014 and December 2016 (Fig 1). Seventy-eight percent (n = 7,688) of these persons were screened just once within the study period, 1,366 (14%) were screened twice and 744 (8%) were screened three or more times within the period (range 3–13 times). Nine percent of all individuals screened (926/9798) had a positive TB screening test results (by either TST or QFT) and were referred to the health department for further evaluation. Among those referred, 777 (84%) presented at the TB clinic for clinical evaluation and 149 persons did not follow up for evaluation. Eight persons among those who attended the referral appointment did not complete the evaluation process. Two of these had sputum samples collected for acid-fast bacilli smear and culture but the patients did not return (and could not be located) after the initial visit, one person signed a refusal for further evaluation and five left the clinic before a clinician could attend them.

Among the 769 persons who completed the evaluation process, 350 (46%) were diagnosed with untreated LTBI and were offered LTBI treatment. A total of 321 (92%) persons accepted to initiate treatment and 29 persons did not. Eighteen (6%) of those who accepted treatment were started on 3HP while 303 (94%) were placed on 4R regimen. Twenty-nine persons among the 4R initiators notified the TB clinic of their relocation from the area during treatment and had their treatment successfully transferred to other jurisdictions. These were excluded from the study, leaving 274 homeless persons in the final study cohort. Sixty-six percent (n = 181) of the 274 were treated by DOT while 93 (34%) were treated by SAT.

### Study cohort

Of the 274 homeless persons who initiated daily rifampin for LTBI treatment at the health department, 89% were men, 93% were African American and 80% were over 39 years old (age range: 22–77 years). About 19% reported current alcohol use, 8% reported a history of illicit drug use and 9% reported living with at least one diagnosed mental health problem ranging from anxiety disorder to schizophrenia (Table 1). Three percent of the cohort was HIV positive. There were statistically significant differences in the gender (p = 0.008), alcohol use (p = 0.001), drug use (p = 0.036) and mental health diagnoses (p = 0.006) distribution of the homeless persons treated by DOT compared to SAT.

**Table 1:** Distribution of baseline characteristics of homeless adults treated for latent tuberculosis infection with four months daily rifampin regimen in Fulton County, Georgia (2014–2016).

Patient characteristics	Total (n = 274) n (%)	DOT (n = 181) n (%)	SAT (n = 93) n (%)	p value^a^
**Gender**				
Male	242 (89)	153 (85)	89 (96)	**0.005**
Female	32 (11)	28 (15)	4 (4)	
**Race**				
Black (African American)	255 (93)	166 (92)	89 (96)	0.316
Not Black/African American	19 (7)	15 (8)	4 (4)	
**Age**				
Age (median (IQR))	52 (15)	52 (15)	51 (14)	
Under 40 years old	55 (20)	37 (20)	18 (19)	0.503
40–49 years old	59 (22)	40 (22)	19 (20)	
50–59 years old	105 (38)	64 (35)	41 (44)	
60 years plus	55 (20)	40 (22)	15 (16)	
**HIV status**^**b**^				
Positive	9 (3)	6 (3)	4 (4)	0.409
Negative	214 (78)	173 (96)	86 (92)	
**Current alcohol use (any quantity)**				
Yes	52 (19)	45 (25)	7 (8)	**0.001**
No	222 (81)	136 (75)	86 (92)	
**Illicit drug use**				
Yes	23 (8)	20 (11)	3 (3)	**0.036**
No	251 (92)	161 (89)	90 (97)	
**Any mental health disorder**				
Yes	24 (9)	22 (12)	2 (2)	**0.006**
No	250 (91)	159 (88)	91 (98)	

^a^Chi-squared test | Statistically significant p values have been bolded for ease of interpretation. |

^b^HIV Status– 2% missing data (n = 5)

### Propensity score estimation and diagnostics

**Fig 2:**
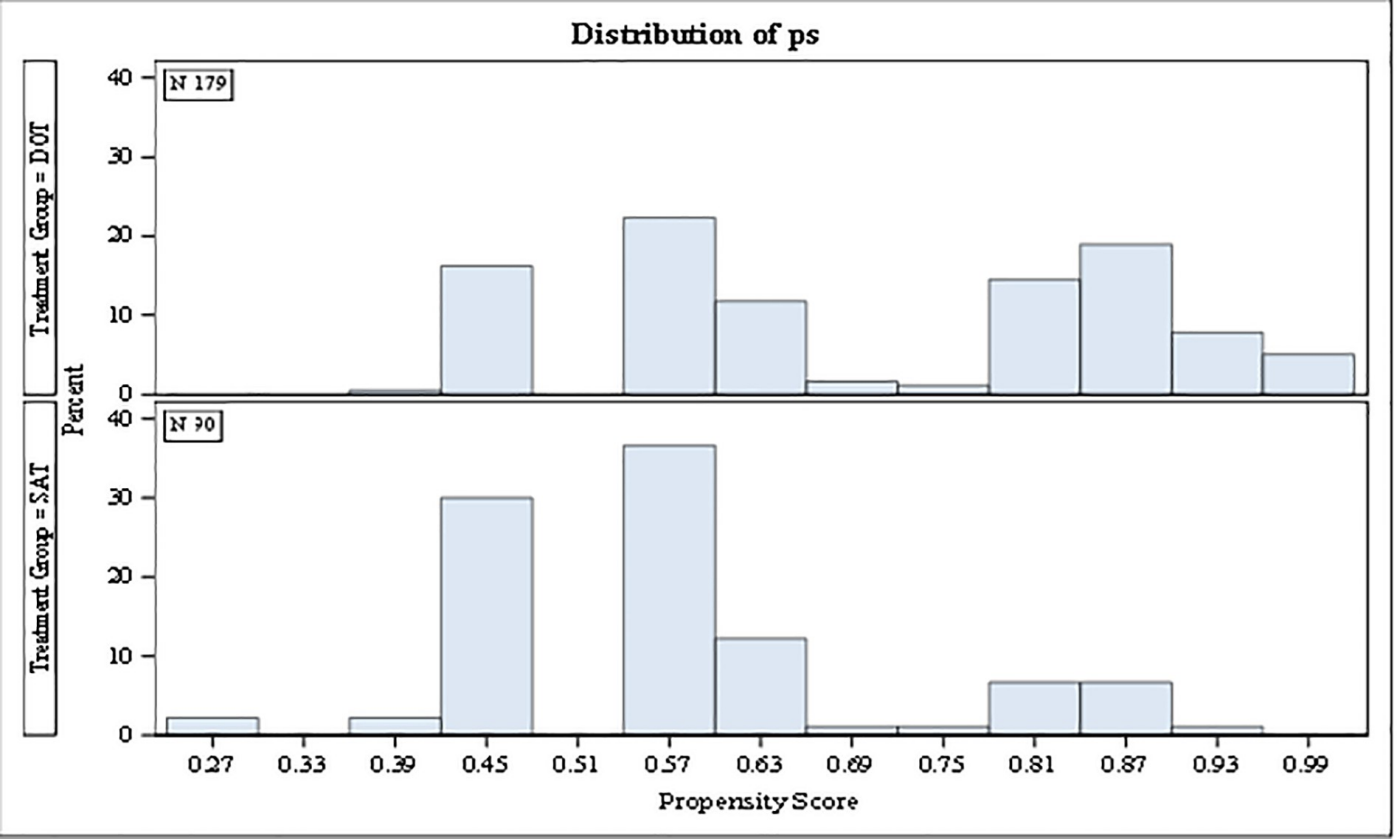
Histogram of propensity score distribution in the LTBI treatment groups.

All measured patient characteristics were included in the propensity score estimation model and histograms comparing the distribution of the estimated propensity scores showed considerable overlap in the spread of the scores in both treatment groups (Fig 2). Details of the covariate balance assessment are shown in Table 2. All measured characteristics were evenly distributed between the treatment groups after weighting with all standardized differences below the bench-mark (0.15).

**Table 2:** Balance assessment using standardized differences.

	Unweighted Sample			IPTW weighted Sample		
	DOT (%)	SAT (%)	Standardized difference	DOT (%)	SAT (%)	*Standardized difference
Male	85	96	-0.38	88	89	-0.03
Black	92	96	-0.17	93	96	-0.13
Under 40 years old	20	19	0.03	20	18	0.05
40–49 years old	22	20	0.05	21	21	0
50–59 years old	35	44	-0.18	39	40	-0.04
60 years plus	22	16	0.15	20	20	-0.02
HIV negative	97	96	0.05	97	98	-0.06
Alcohol use (Yes)	25	8	0.47	19	16	0.08
Illicit drug use (Yes)	11	3	0.32	9	9	0
Mental illness (Yes)	12	2	0.40	9	6	0.11

*Balance achieved (shown by decreased standardized differences) in distribution of covariates with large differences at baseline after propensity score weighting.

### LTBI treatment completion and bivariate analysis

Overall, 65% of the all 274 rifampin initiators (unweighted sample) completed LTBI treatment and a slightly higher proportion of DOT-treated patients (65%) than SAT-treated patients (63%) completed treatment. More alcohol users treated by DOT (62%) completed LTBI treatment than those treated by SAT (43%). In the (IPTW) weighted sample, treatment completion among the DOT-treated group was 68% while completion among the SAT-treated group was 62% (Table 3).

**Table 3:** Latent TB infection treatment completion in the unweighted and weighted samples of homeless persons treated with 4 months of daily rifampin in Fulton County, Georgia (2014–2016).

	Unweighted sample				(IPTW) weighted sample			
	DOT^a^		SAT^a^		DOT^b^		SAT^b^	
	Incomplete (%)	Completed (%)	Incomplete (%)	Completed (%)	Incomplete (%)	Completed (%)	Incomplete (%)	Completed (%)
DOT	35	65	-	-	32	68	-	-
SAT	-	-	37	63	-	-	38	62
**Gender**								
Female	50	50	50	50	50	50	62	38
Male	32	68	36	64	30	70	35	65
**Race**								
Black	34	66	36	64	31	69	38	62
Non-black	47	53	50	50	48	52	41	59
**Age (years)**								
< 40 yrs. old	49	51	44	56	47	53	40	60
40–49 yrs. old	35	65	42	58	34	66	42	58
50–59 yrs. old	30	70	34	66	26	74	40	60
60 yrs. plus	30	70	27	73	29	71	30	70
**HIV status**								
Negative	34	66	36	64	32	68	39	61
Positive	50	50	0	100	42	58	0	100
**Alcohol use**								
No	34	66	35	65	31	69	37	63
Yes	38	62	57	43	38	62	44	56
**Illicit drug use**								
No	34	66	37	63	32	68	39	61
Yes	40	60	33	67	41	59	34	66
**Mental health**								
No	35	65	36	64	32	68	38	62
Yes	32	68	50	50	33	67	48	52

^a^-Actual counts in unweighted sample.

^b^-Count estimates from IPTW-weighted sample (Pseudo-sample: Counts may not add up to total treated).

Details of bivariate analysis (unweighted sample) are shown in Table 4. Unadjusted analysis of the unweighted sample showed a small, non-significant improvement in treatment completion among the DOT-treated group compared to the SAT-treated group [unadjusted odds ratio (95% CI), cOR: 1.08 (0.64, 1.82), p = 0.774]. Among all covariates explored in unadjusted analysis, age between 50 and 59 years was the only patient characteristic marginally associated with treatment completion [cOR: 1.08 (1.96 (1.00, 3.83), p = 0.05].

**Table 4:** Bivariate associations between patient characteristics at baseline and treatment completion (Unweighted sample) among 274 homeless persons on 4 months rifampin for latent TB infection treatment in Fulton County, GA (2014–2016).

	Crude OR (95% CI)	p value
**Treatment type**		
DOT	1.08 (0.64, 1.82)	0.774
SAT	*ref*	
**Gender**		
Male	1.99 (0.95, 4.18)	0.070
Female	*ref*	
**Race**		
Black	1.71 (0.67, 4.36)	0.263
Non-Black	*ref*	
**Age (years)**		
<40	*ref*	
40–49	1.51 (0.71, 3.18)	0.282
50–59	1.96 (1.00, 3.83)	0.050
60 plus	2.19 (1.00, 4.80)	0.051
**HIV status**		
Positive	1.24 (0.31, 4.92)	0.757
Negative	*ref*	
**Reported Alcohol use**		
Yes	0.77 (0.41, 1.43)	0.405
No	*ref*	
**Reported Illicit drug use**		
Yes	0.84 (0.35, 2.02)	0.696
No	*ref*	
**Reported prior mental health diagnoses**		
Yes	1.11 (0.46, 2.69)	0.825
No	*ref*	

### Estimation of treatment effect

Adjusted analysis (Table 5) with the parsimonious, IPTW-weighted model showed that DOT significantly improved the odds of LTBI treatment completion in the sample by 30% compared to SAT ([Odds ratio (95% CI), OR: 1.30 (1.01, 1.67), p = 0.045]. Addition of all measured covariates in the doubly robust model (fully weighted and adjusted model) showed similar effect of DOT on treatment completion [OR (95% CI): 1.40 (1.07, 1.82), p = 0.014]. In this fully adjusted and weighted model, male gender, black race, age (50–59, 60 years and older) and positive HIV status were also significantly associated with increased odds of treatment completion while alcohol use was associated with decreased odds of completing treatment [OR (95% CI): 1.51 (0.36, 0.74)].

**Table 5:** Adjusted odds ratios of LTBI treatment completion among 274 homeless adults treated with 4 months of daily rifampin in Fulton County, Georgia (2014–2016).

	Weighted model (Parsimonious)^a^	p value	Adjusted and Weighted model^b^	p value
	OR (95% CI)		OR (95% CI)	
**Treatment type**	** **		** **	
DOT	1.30 (1.01, 1.67)	**0.045**	1.40 (1.07, 1.82)	**0.014**
SAT	*ref*		*ref*	
**Gender**				
Male	-		3.40 (2.32, 4.98)	**< .0001**
Female			*ref*	
**Race**				
Black	-		1.75 (1.02, 2.99)	**0.042**
Non-Black			*ref*	
**Age (years)**				
<40	-		*ref*	
40–49	-		1.36 (0.92, 2.01)	0.119
50–59	-		1.74 (1.22, 2.47)	**0.002**
60 plus	-		2.37 (1.57, 3.80)	**< .0001**
**HIV status**				
Positive	-		3.50 (1.30, 9.38)	**0.013**
Negative			*ref*	
**Alcohol use**				
Yes	-		0.51 (0.36, 0.74)	**0.0003**
No			*ref*	
**Illicit drug use**				
Yes	-		1.34 (0.81, 2.23)	0.258
No			*ref*	
**Mental health diagnoses**				
Yes	-		0.70 (0.42, 1.15)	0.157
No			*ref*	

Note: Statistically significant p values have been bolded for ease of interpretation.

^a^. Parsimonious model (IPTW-weighted logistic regression model with treatment type as the only covariate specified).

^b^. Doubly robust model (IPTW-weighted model with all measured covariates in addition to the main exposure (treatment type)).

## Discussion

Detection and treatment of LTBI among homeless persons is of immense public health importance in achieving the goal of TB elimination in the United States[3,5]. The presence of unaddressed co-morbidities (mental health-related and non-mental health-related), substance addictions (alcoholic and non-alcoholic) and poor access to medical care make effective treatment of LTBI in these persons difficult[11]. These factors in combination with poor adherence to LTBI treatment make the homeless population a fertile ground for frequent TB outbreaks.

In this retrospective study, about one third of the homeless persons who initiated rifampin for LTBI treatment completed their prescribed treatment. This compares favorably to the findings from the published studies on LTBI treatment adherence among homeless persons[18,35–38]. Goswami *et al* in their study reported treatment completion rate of 53% among homeless persons who initiated LTBI treatment while Nyamathi *et al* reported at overall completion rate of 51% among 520 homeless adults who initiated treatment for LTBI. The study by Tulsky *et al* reported a higher treatment completion (86%) in their randomized controlled trial on LTBI treatment among homeless persons in California.[36] However, only 49% of their study subjects were reported to be current residents of an emergency shelter at the time of treatment initiation[36] In comparison, all the subjects included in this study were current residents of an emergency shelter at the time of LTBI treatment initiation indicating a higher concentration of the target population in this study than in the study by Tulsky *et al*.

Propensity score analysis was employed in the assessment of the effect of treatment modality used on treatment completion. This was done primarily because there were statistically significant and clinically meaningful differences between the distributions of patient characteristics in the two treatment groups at treatment initiation. Patients who were treated by SAT were more likely to be men, non-reporters of alcohol or illicit drug use and have no history of previously diagnosed mental health disorder at treatment initiation than those treated by SAT. Appropriately specified propensity scores simulate the conditions obtained in randomized controlled trials, the gold standard of epidemiologic studies, and is usually used to achieve exchangeability of exposure groups in observational studies.[24,27,30,32,39] Examination of the spread of the estimated propensity scores showed considerable overlap in the distribution of the scores in both treatment groups indication appropriate specification of the propensity score estimation model. We also saw considerable reduction in the standardized differences between the covariate distributions in the weighted sample further indicating that balance in baseline distribution of the measured patent characteristics was achieved and the underlying propensity of treatment used for each subject in the cohort was appropriately specified. [24,27,30,39]

One of the core benefits of using propensity score based models to estimate treatment effect is the ability to model outcome using only one covariate (the main exposure) in a parsimonious model as against including multiple covariates in the effect estimate model in the traditional multivariate models. However, other variations to this have been used in scientific literature and this includes adding relevant variables in the propensity score outcome model to yield a model that is both fully adjusted and propensity score weighted (doubly robust) outcome model. [24,27,32–34] Both approaches (parsimonious and doubly robust models) were used in this study to examine the effect of using DOT versus SAT in treating homeless persons for latent TB infection. Both outcome models showed that the use of DOT for LTBI treatment resulted in significant improvement in treatment completion among the homeless persons compared to SAT. SAT is the current standard of practice for LTBI treatment in the general population. However, several issues come into play when this modality is employed in LTBI treatment among homeless persons These issues include potential lack of appropriate medication storage environment, possibility of pill box loss or theft, possibility of exchanging the medications for money or other necessities and often simple forgetfulness in the pursuit of more urgent survival needs. We are not aware of any previous study focused solely on LTBI treatment adherence in homeless persons on daily rifampin treatment, however previous studies on DOT and LTBI treatment in the general population and other high-risk groups have all reported increased adherence on DOT.[15,16,40] Our study provides support to the use of this adherence-improving intervention for homeless persons on daily rifampin regimen for LTBI treatment.

Nearly one fifth of the study sample reported alcohol use at the time of treatment initiation and a higher proportion of the self-reported alcohol users treated by DOT in both the unweighted and weighted samples completed the prescribed LTBI treatment compared to the alcohol users treated by SAT. Estimation of effect in the doubly robust model showed that persons who reported alcohol use were less likely to complete treatment compared to those who did not report any alcohol use. Regular use of alcohol and/or alcohol dependence, conditions quite common among homeless persons, do not only constitute strong risk factors for LTBI progression to active TB disease [3], but are also deterrents to treatment adherence among homeless persons.[15] This benefit of improvement in treatment adherence among homeless alcohol users treated by DOT may justify its use in LTBI treatment to both reduce risk of progression to TB disease and improve the likelihood of treatment completion.

## Limitations

Our study had some limitations. Data used for this analysis were abstracted from routine clinical care records and therefore, relied heavily on the accuracy and completeness of the clinical care notes. Social characteristics (alcohol use illicit drug use and mental health diagnoses) examined were self-reported findings noted in the clinical records of patient-clinician encounters and thus depended both on the thoroughness of the clinician in inquiring about and recording these responses and accuracy of the patient’s response. In addition, there may have been social desirability bias influencing the patient responses to inquiries about alcohol and drug use and the impact of this bias on the outcome was not examined. The DOT regimen used in this study was administered five days a week. There is no published data on the efficacy of rifampin given five days a week in latent tuberculosis treatment. This dosing regimen was used given the prevailing circumstances at the time.[22]

This study would have benefitted from moderation analyses to assess the consistency of treatment effect across various subgroups of homeless persons treated for LTBI. Previous studies that have combined propensity score analyses with moderation analyses show that while it is more ideal to use subgroup-specific propensity scores for subgroup analyses (rather than propensity scores estimated in the full cohort), correctly specified propensity scores estimated in the full cohort remain valid within subgroups as long as the scores accurately reflect the underlying propensity of treatment for each subject and the subgroups are of sufficient size.[27,31–34,41] Our study sample did not have sufficient sample power at the sub-group level to allow robust estimation of treatment effect in moderation analyses and we suggest future research on this as an improvement of the findings on this study.

## Public health implications

As the march towards TB elimination in the United States progresses, stronger attention will need to be paid to the detection and treatment of latent TB among high-risk populations like persons who reside in emergency homeless shelters and transitional housing facilities. Given the low levels of treatment acceptance, adherence and completion seen in previous years in this population, it is imperative now to seek more effective means of improving LTBI treatment adherence and completion among homeless persons and decrease risk of progression to active disease and emergence of TB outbreaks. Our study provides the statistical evidence to support the use of DOT to treat LTBI in unstably-housed persons in urban cities within the United States. As a next step, we suggest that the potential for achieving similar improvement in LTBI treatment adherence as observed in our study with in-person DOT needs to be evaluated using video DOT due to its’ attractive benefit of reduced cost of implementation and manpower, particularly for rural areas where in-person DOT might be difficult to implement.

## Supporting information

S1 File Study dataset including codebook. (XLSX)
